# Tumour necrosis factor-α and adenosine in endotoxin shockleading related cardiovascular symptoms

**DOI:** 10.1155/S0962935195000731

**Published:** 1995-11

**Authors:** S. Sipka, T. Seres, Z. Dinya, Z. Szekanecz, J. Szentmiklósi, E. Bodolay, G. Szegedi

**Affiliations:** 1 Third Department of Medicine University Mdical School Hungary; 2 Department of Pharmacology University Mdical School Hungary; 3 Department of Orgaic Chemistry Lajos Kossuth University Debrecen Hungary

## Abstract

We have observed uncontrollable cardiogenic shock as a cardiovascular manifestation of systemic inflammatory response syndrome (SIRS) leading to death in a 62-year-old woman. The diagnosis of SIRS was based on the demonstration of endotoxinaemia, and highly elevated plasma levels of tumour necrosis factor (TNF)-α, and interleukin (IL)-10. We suggest that these cytokines may contribute to the terminal SIRS-related arrythmias, impaired myocardial contractility, as well as increased vascular permeability. In addition, the increased production of adenosine, a counter-regulatory mediator of inflammation, may also play a role in cardiodepression. We suggest a relationship between the action of TNF-α , IL-10 and adenosine in the pathogenesis of circulatory symptoms described above.


Mediators of Inflammation 4, 454–455 (1995)

### Tumour necrosis factor-α and adenosine in endotoxin shock-related cardiovascular symptoms

S. Sipka,[1,CA] T. Seres,[1] Z. Dinya,[3] Z. Szekanecz,[1] J. Szentmiklósi,[2] E. Bodolay[1] and G. Szegedi[1]

[1]Third Department of Medicine and [2]Department of Pharmacology, University Medical School, and [3]Department of Organic Chemistry, Lajos Kossuth University, Debrecen, Hungary

[CA]Corresponding Author

**We have observed uncontrollable cardiogenic shock as a cardiovascular manifestation of systemic inflammatory response syndrome (SIRS) leading to death in a 62-year-old woman. The diagnosis of SIRS was based on the demonstration of endotoxinaemia, and highly elevated plasma levels of tumour necrosis factor (TNF)-α, and interleukin (IL)-10. We suggest that these cytokines may contribute to the terminal SIRS-related arrythmias, impaired myocardial contractility, as well as increased vascular permeability. In addition, the increased production of adenosine, a counter-regulatory mediator of inflammation, may also play a role in cardiodepression. We suggest a relationship between the action of TNF-α, IL-10 and adenosine in the pathogenesis of circulatory symptoms described above.**

**Key words:** Adenosine, Endotoxin shock, Tumour necrosis factor-α

### Introduction

A number of inflammatory mediators may be involved in the pathogenesis of endotoxin-induced systemic inflammatory response syndrome (SIRS). The most severe stage of SIRS is the multiple organ dysfunction syndrome (MODS), which affects, among others, the cardiovascular system and this leads to death in half of these patients.[1,2] The cascade of events in SIRS may be triggered by endotoxin, which leads to the activation of monocytes/macrophages. These cells begin producing high amounts of tumour necrosis factor (TNF)-α and interleukin (IL)-1; followed by the secretion of other mediators, such as IL-6, IL-8, platelet-activating factor (PAF), leukotrienes, endothelin-1, and others.[3] TNF-α and IL-1 may also stimulate T cells to produce IL-2. Increasing amounts of data suggest a pathogenetic relationship between these mediators in SIRS-related symptoms.[3] Adenosine is a well-known negative chronotropic, inotropic and dromotropic agent[4] and its production by phagocytes can be stimulated by hypoxia[5–8] or other mediators, such as PAF.[9] Thus, adenosine may also be involved in SIRS.[10–11] By presenting a case history, we wished to demonstrate the possible pathogenetic role of certain cytokines, such as TNF-α and IL-10, as well as the counter-regulatory mediator adenosine in the cardiovascular events underlying SIRS.

### Case history

A 62-year-old woman was admitted to our institution with a 1-year history of fever, anaemia, fatigue and splenomegaly. The patient had previously been examined at a number of hospitals, where increased erythrocyte sedimentation rate and sideropenic anaemia with low iron binding capacity were found. Other laboratory values were normal. Several diagnostic tests had been performed throughout the months without any direct evidence for infectious, immunopathological diseases or malignancies. However, we observed a 38–39°C fever and other clinical symptoms of endotoxin shock, which in a few days led to severe generalized oedema together with the signs of multiorgan failure (MODS). This latter involved the lungs, the cardiovascular system, the kidneys and, terminally, the central nervous system. All serial blood culture and serological tests were negative for bacteria, viruses and fungi. However, the plasma level of endotoxin was increased (3.85 IU/ml) compared to normal plasma (< 1 IU/ml) as determined by the Limulus assay (Biomondex, Budapest) (Table 1). In addition, the diagnosis of SIRS was also based on the clinical and laboratory signs of MODS and diffuse intravascular coagulation. Immunological laboratory techniques showed complement activation, higher IgM level and decreased *in vitro* chemotaxis of neutrophils and monocytes. Terminally, the patient had impaired myocardial contractility associated with atrial fibrillation, catecholamine-resistant cardiogenic shock and died of asystolia. Autopsy showed the hypoxic damage of most organs, again with no macroscopic signs of tumours or inflammation.

Plasma samples were taken from the patient 1 day before her death. This was the time when

© 1995 Rapid Science Publishers

**Table 1.** Cytokine and adenosine levels in plasma

| | Sample | Reference |
|---|---|---|
| TNF-α | 126 pg/ml | < 15.7 pg/ml |
| IL-2 | N.D.* | < 31.3 pg/ml |
| IL-10 | 250 pg/ml | < 15.6 pg/ml |
| Adenosine | 0.75 μmol/l | < 0.12 μmol/l |
| Endotoxin | 3.85 IU/ml | < 1 IU/ml |

*N.D. = Non-detectable.

the most serious cardiovascular events described above, as well as generalized oedema were observed. Samples were assayed for TNF-α, IL-2 (Amersham) and IL-10 (Biosource) using ELISA systems. Plasma adenosine concentration was determined by mass spectroscopy.[4] Results are given in Table 1. IL-10, TNF-α and adenosine showed striking 16-, 8- and 6-fold increases above the indicated reference level (normal controls), respectively. In contrast, we could not detect any IL-2 in the plasma.

### Discussion

The high amount of TNF-α found in our patient's plasma may be important in the pathologic regulation underlying SIRS as it is the first mediator showing increased production by macrophages.[3] As TNF-α itself may cause endothelial injury and leak, systemic vasodilatation, and decreases in myocardial contractility,[3] it may, at least in part, be responsible for the impaired myocardial function and generalized oedema. TNF-α stimulates the production of IL-1, IL-6, IL-8 and PAF, as well as the metabolism of arachidonic acid. Among these mediators leukotrienes and PAF may act in concert with endotoxin and TNF-α in increasing capillary permeability.[3] In the case of our patient, therapy included pentoxyphylline and high-dose glucocorticoids, drugs known to suppress cytokine production. Although no fever was observed in the last week prior to death, probably due to the effects of this combined treatment, TNF-α production could not be totally inhibited, which resulted in the further perpetuation of SIRS and death.

Elevated adenosine levels were also found in our patient's plasma. As described above, adenosine production can be induced by hypoxia,[5–8] as well as PAF or LTB4,[9] pathogenetic factors also important in the pathogenesis of SIRS.[3] Adenosine can also act as a counter-regulatory mediator by inhibiting the production of TNF-α.[12] Its negative effects on the myocardium described above,[4] however, can be fatal under certain conditions, as it was in the case of our patient.

The enormously high amount of plasma IL-10 measured could be secreted mainly by Th2 type T cells and it could take part in processes suppressing TNF-α and IL-2 production.[13] The reduction of TNF-α production is a mode of action of IL-10 that diminishes mortality to lethal endotoxinaemia in mice.[14]

Our concept, suggesting the role of certain cytokines in endotoxin shock-related heart failure, may help to explain some aspects of cardiac mortality in SIRS. However, other inflammatory mediators, such as adenosine may also regulate the action of these cytokines. At the same time they may act independently from cytokines and directly cause cardiodepression. In addition, we suggest that TNF-α or adenosine antagonists, such as pentoxifylline or theophylline, respectively, may be used in the therapy of SIRS and the related MODS. Future therapeutic tools may include monoclonal antibodies raised against these mediators or antiinflammatory cytokines, such as IL-10.[14]

### References

1. ACCP/SCCM Consensus Conference. Definition for sepsis and organ failure and guidelines for the use of innovative therapies in sepsis. *Crit Care Med* 1992; **20:** 864–870.
2. Parrillo JE. Pathogenetic mechanisms of septic shock. *N Engl J Med* 1993; **328:** 1471–1477.
3. Giroir BP. Mediators of septic shock: new approaches for interrupting the endogenous inflammatory cascade. *Crit Care Med* 1993; **21:** 780–789.
4. Evans DB, Schenden JA, Bristol JA. Adenosine receptors mediating cardiac depression. *Life Sci* 1982; **31:** 2425–2432.
5. Engler R. Granulocytes and oxidative injury in myocardial ischemia and reperfusion. *Fed Proc* 1987; **46:** 2395–2396.
6. McCord MJ. Oxygen-derived radicals: a link between reperfusion injury and inflammation. *Fed Proc* 1987; **46:** 2402–2406.
7. Gorman MW, He MX, Sparks HV. Adenosine formation during hypoxia in isolated hearts: effect of adrenergic blockade. *J Mol Cell Cardiol* 1994; **26:** 1613–1623.
8. Thomas T, Marshall JM. Interdependence of respiratory and cardiovascular changes induced by systemic hypoxia in the rats: the roles of adenosine. *J Physiol* 1994; **480:** 627–636.
9. Sipka S, Dinya Z, Koltai M, Braquet P, Szegedi G. Release of adenosine from human neutrophils stimulated by platelet activating factor, leukotriene $B_4$ and opsonized zymosan. *Mediators Inflamm* 1992; **1:** 267–271.
10. Adnot S, Lefort J, Vargaftig B. PAF, endotoxinaemia and ginkgolides. In: Braquet P, ed. *Ginkgolides—Chemistry, Biology, Pharmacology and Clinical Perspectives. Volume 1.* Barcelona: J.R. Prous Science Publishers, 1989; 453–456.
11. Riedel A, Meyer E, Heinroth-Hoffmann I, Mentz P, Mentz G, Mest H-J. BN52021 abolishes PAF-induced cardiac rhythm disturbances. In: Braquet P, ed. *Ginkgolides—Chemistry, Biology, Pharmacology and Clinical Perspectives, Volume 2.* Barcelona: J.R. Prous Science Publishers, 1989; 275–289.
12. Reinstein LJ, Lichtman SN, Currin RT, Wang J, Thurman RG, Lemastes JJ. Suppression of lipopolysaccharide-induced release of tumor necrosis factor by adenosine: evidence for A2 receptors on rat Kupffer cells. *Hepatology* 1994; **19:** 1445–1452.
13. Moore KW, O'Garra A, De Waal Malefyt R, Vieira P, Mosmann TR. Interleukin-10. *Annu Rev Immunol* 1993; **11:** 165–190.
14. Rogy MA, Auffenberg T, Espat NJ *et al.* Human tumor necrosis factor receptor (p55) and interleukin 10 gene transfer in the mouse reduces mortality to lethal endotoxinaemia and also attenuates local inflammatory responses. *J Exp Med* 1995; **181:** 2289–2293.

**Received 6 June 1995;**
**accepted in final form 6 September 1995**
